# Disruption of Small GTPase Rab7 Exacerbates the Severity of Acute Pancreatitis in Experimental Mouse Models

**DOI:** 10.1038/s41598-017-02988-3

**Published:** 2017-06-06

**Authors:** Kenichi Takahashi, Hirosato Mashima, Kouichi Miura, Daichi Maeda, Akiteru Goto, Takashi Goto, Ge-Hong Sun-Wada, Yoh Wada, Hirohide Ohnishi

**Affiliations:** 1 Department of Gastroenterology and Hepato-Biliary-Pancreatology, Akita University Graduate School of Medicine, Akita, Japan; 2 Department of Cellular and Organ Pathology, Akita University Graduate School of Medicine, Akita, Japan; 3 Department of Gastroenterology, Saitama Medical Center, Jichi Medical University, Saitama, Japan; 4 Department of Biochemistry, Faculty of Pharmaceutical Sciences, Doshisha Women’s College, Kyoto, Japan; 5 Division of Biological Science, Institute of Scientific and Industrial Research, Osaka University, Osaka, Japan; 6 Japan Organization of Occupational Health and Safety, Kanagawa, Japan

## Abstract

Although aberrations of intracellular vesicle transport systems towards lysosomes including autophagy and endocytosis are involved in the onset and progression of acute pancreatitis, the molecular mechanisms underlying such aberrations remain unclear. The pathways of autophagy and endocytosis are closely related, and Rab7 plays crucial roles in both. In this study, we analyzed the function of Rab7 in acute pancreatitis using pancreas-specific Rab7 knockout (Rab7^Δpan^) mice. In Rab7^Δpan^ pancreatic acinar cells, the maturation steps of both endosomes and autophagosomes were deteriorated, and the lysosomal functions were affected. In experimental models of acute pancreatitis, the histopathological severity, serum amylase concentration and intra-pancreatic trypsin activity were significantly higher in Rab7^Δpan^ mice than in wild-type mice. Furthermore, the autophagy process was blocked in Rab7^Δpan^ pancreas compared with wild-type mice. In addition, larger autophagic vacuoles that colocalize with early endosome antigen 1 (EEA1) but not with lysosomal-associated membrane protein (LAMP)-1 were much more frequently formed in Rab7^Δpan^ pancreatic acinar cells. Accordingly, Rab7 deficiency exacerbates the severity of acute pancreatitis by impairing the autophagic and endocytic pathways toward lysosomes.

## Introduction

Acute pancreatitis develops primarily due to pancreatic acinar cell damage, including cell death from heavy alcohol use and can be lethal in some cases despite various intensive treatments. Although the molecular mechanism of the onset and progression of acute pancreatitis have been extensively studied, the precise mechanism underlying this disease remains unclear. The representative pathological features observed in pancreatic acinar cells at the initiation phase of acute pancreatitis are the formation of vacuoles in the cytoplasm and intracellular trypsin activation.

Autophagy is an intracellular vesicle trafficking process used to degrade old cytoplasmic materials and compartments in the lysosomal system^1^. Autophagy is essential for maintaining cellular homeostasis and also helps ensure the cellular survival under stressful conditions, such as starvation^2^. In addition, autophagy has been associated with various diseases^3, 4^. To date, three distinct pathways of autophagy are known: macroautophagy, chaperone-mediated autophagy and microautophagy^5^. Of these, the intracellular process of macroautophagy has been most intensively studied and elucidated. Thus, “autophagy” generally refers to macroautophagy, and it is used as such in the present study. In the autophagic process, intracellular substances that should be degraded are first surrounded by phagophores to form autophagosomes. Autophagosomes then maturate to autolysosomes by fusing with lysosomes abundant with various proteases. Finally, the substances enveloped by autolysosomes are degraded by proteases such as cathepsins^1^.

Endocytosis towards lysosomes is another intracellular vesicle trafficking system involved mainly in importing extracellular materials necessary for maintaining cellular homeostasis^6^. Autophagy and endocytosis are closely related. For instance, late endosomes fuse to autophagosomes during their maturation^7^. Furthermore, both processes require lysosomal fusion at their final steps.

It has recently been reported that autophagy is involved in the onset and progression of acute pancreatitis^4, 8^. Furthermore, vacuole formation and trypsin activation in pancreatic acinar cells during acute pancreatitis are presumed to be tightly related to autophagy and lysosomal enzymes^8^. In addition, it has been recently shown that endocytosis in pancreatic acinar cells is also involved in the onset of acute pancreatitis^9^. However, the precise roles of autophagy and endocytosis in acute pancreatitis remain unclear^10–12^.

Rab proteins belongs to the Ras-related GTP-binding protein family and function in various intracellular vesicle trafficking systems including autophagy and endocytosis^6, 13^. Rab7 plays a particularly pivotal role at the late steps of both autophagy and endocytosis and in lysosome biogenesis^7, 14, 15^. We and others have reported that multiple Rab proteins function in diverse vesicle trafficking systems in pancreatic acinar cells^16–19^. Therefore, to elucidate the molecular mechanism underlying the participation of autophagy and endocytosis towards lysosomes in the pathophysiology of acute pancreatitis, we investigated the role of Rab7 in several physiological conditions and acute pancreatitis using pancreas-specific Rab7-deficient mice.

## Results

### Generation of pancreas-specific Rab7-deficient mice

**Figure 1:**
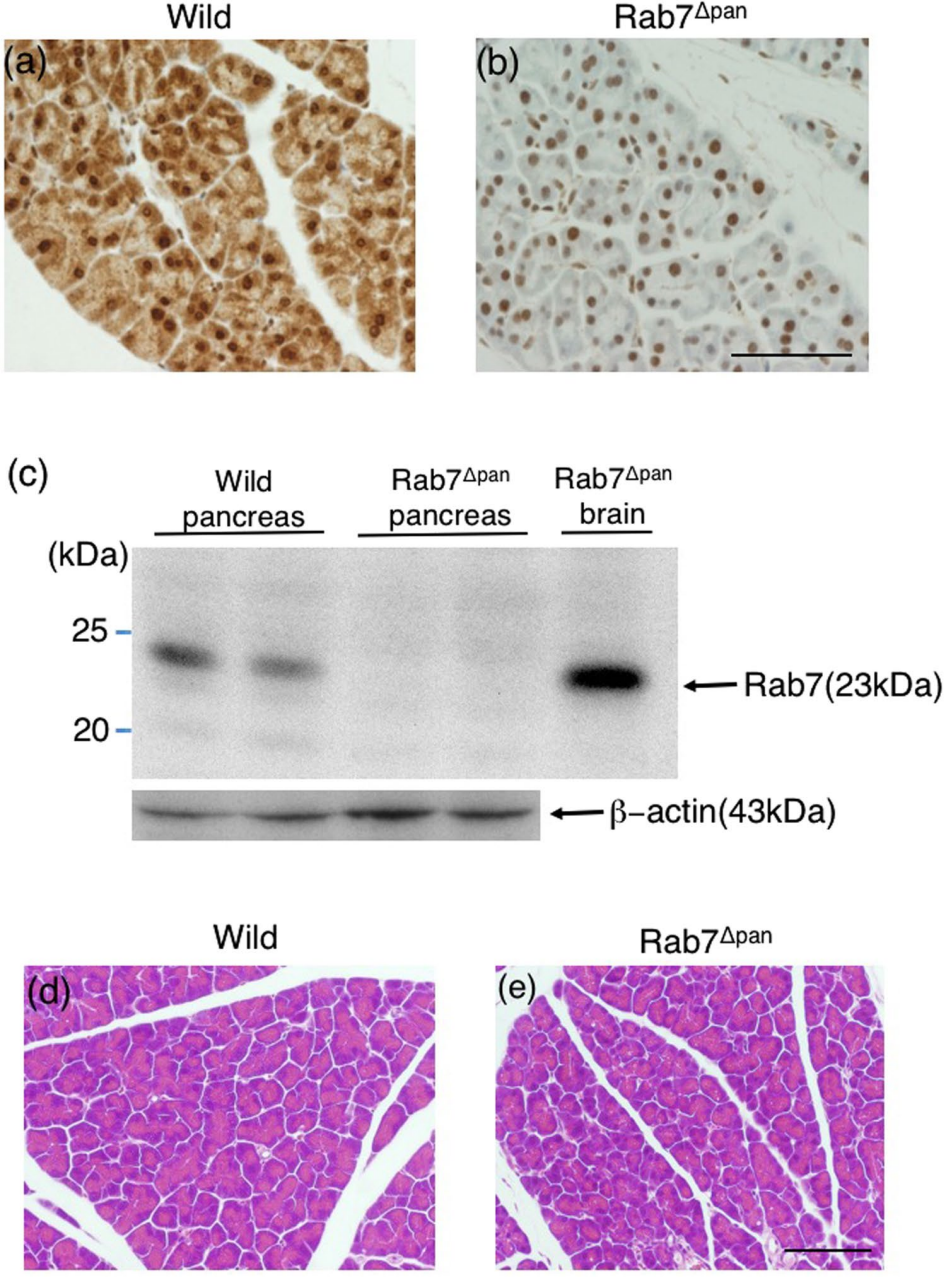
Disruption of Rab7 in Rab7^Δpan^ mouse pancreas. (**a**,**b**) IHC using anti-Rab7 antibody of wild (**a**) and Rab7^Δpan^ (**b**) mouse pancreases. Scale Bar: 50 µm. (**c**) WB of Rab7 using total tissue homogenates of Rab7^Δpan^ pancreas and brain and wild mouse pancreas. (**d**,**e**) HE staining of pancreases of control and Rab7^Δpan^ mice. Scale Bar: 50 µm.

In order to investigate Rab7 participation in acute pancreatitis, we generated pancreas-specific Rab7-deficient (Rab7^Δpan^) mice by crossing Rab7^flox/flox^ mice and Ptf1a-Cre mice. Immunohistochemistry using anti-Rab7 antibody revealed that Rab7 was expressed in pancreatic acinar cells of wild mice (Fig. 1a). In contrast, no apparent signal of Rab7, except for non-specific nuclear staining, was observed in pancreatic acinar cells of Rab7^Δpan^ mice (Fig. 1b). Western blotting (WB) of whole-tissue lysates of the pancreas and brain of Rab7^Δpan^ mice and of wild mice pancreas confirmed that Rab7 was disrupted specifically in Rab7^Δpan^ pancreas (Fig. 1c). Rab7^Δpan^ mice had normal development, and a histological analysis with HE staining showed no histological abnormalities in the Rab7^Δpan^ mouse pancreas compared to that of wild mice (Fig. 1d and e).

### Effect of starvation on autophagy in pancreatic acinar cells of Rab7^Δpan^ mice

**Figure 2:**
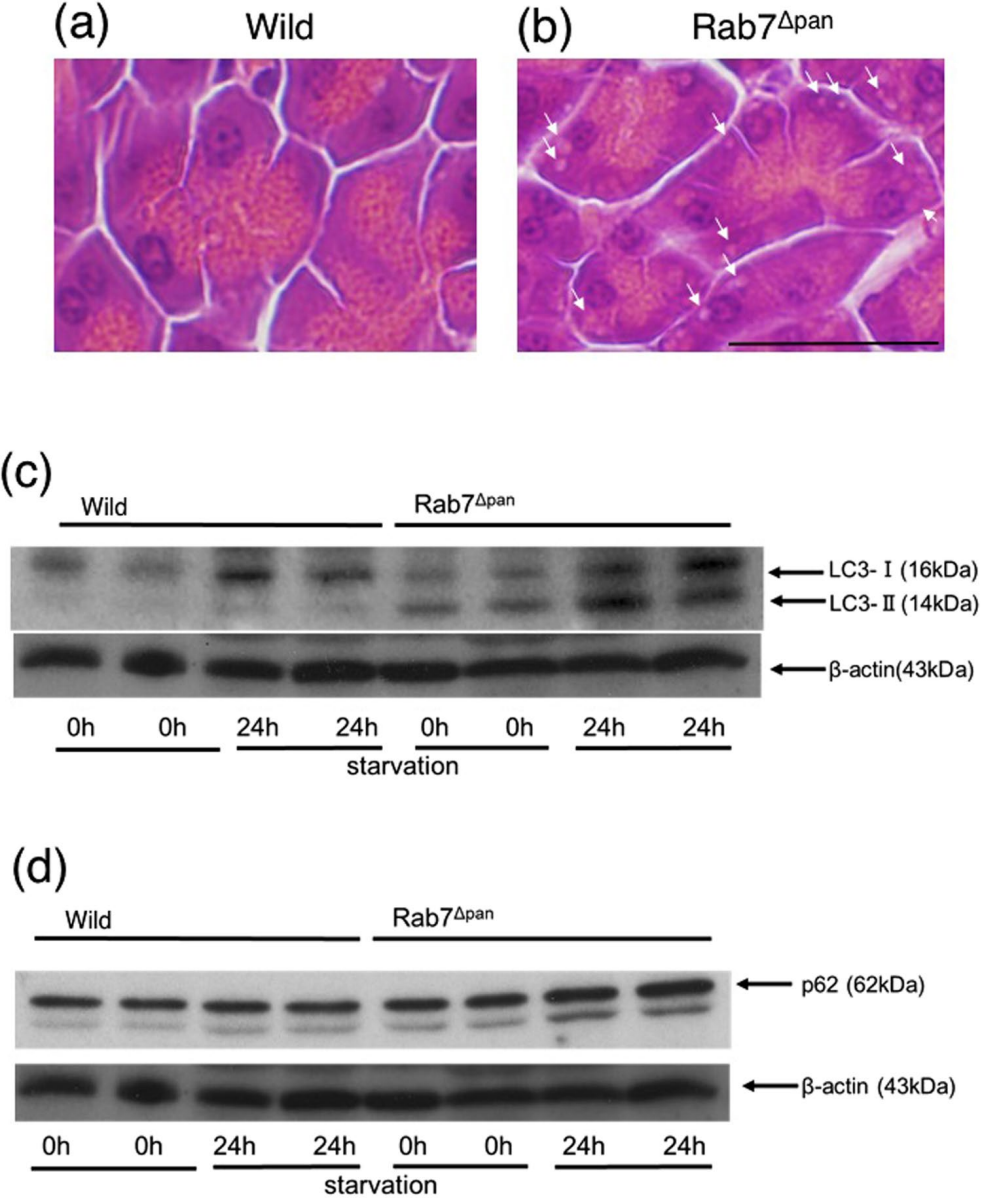
Alteration of autophagy in Rab7^Δpan^ pancreas. (**a**,**b**) HE staining of the pancreas after 24-h starvation showed vacuole formation in Rab7^Δpan^ (**b**) arrows) but not in the wild mouse pancreas (**a**). Scale Bar: 20 µm. (**c**,**d**) WB of LC3 (**c**) and p62 (**d**) using total pancreas homogenates. β-actin was used as an internal loading control. The blots are the representative of two independent experiments with similar results.

We then examined the effect of Rab7 disruption on the autophagic process in pancreatic acinar cells. Autophagy was triggered by 24 h starvation. Although no morphological alteration was observed in wild mouse pancreas after 24 h starvation (Fig. 2a), multiple vacuoles were observed in Rab7^Δpan^ mouse pancreatic acinar cells (Fig. 2b). These data suggest that the autophagic process is aberrant in Rab7^Δpan^ mouse pancreatic acinar cells.

**Figure 3:**
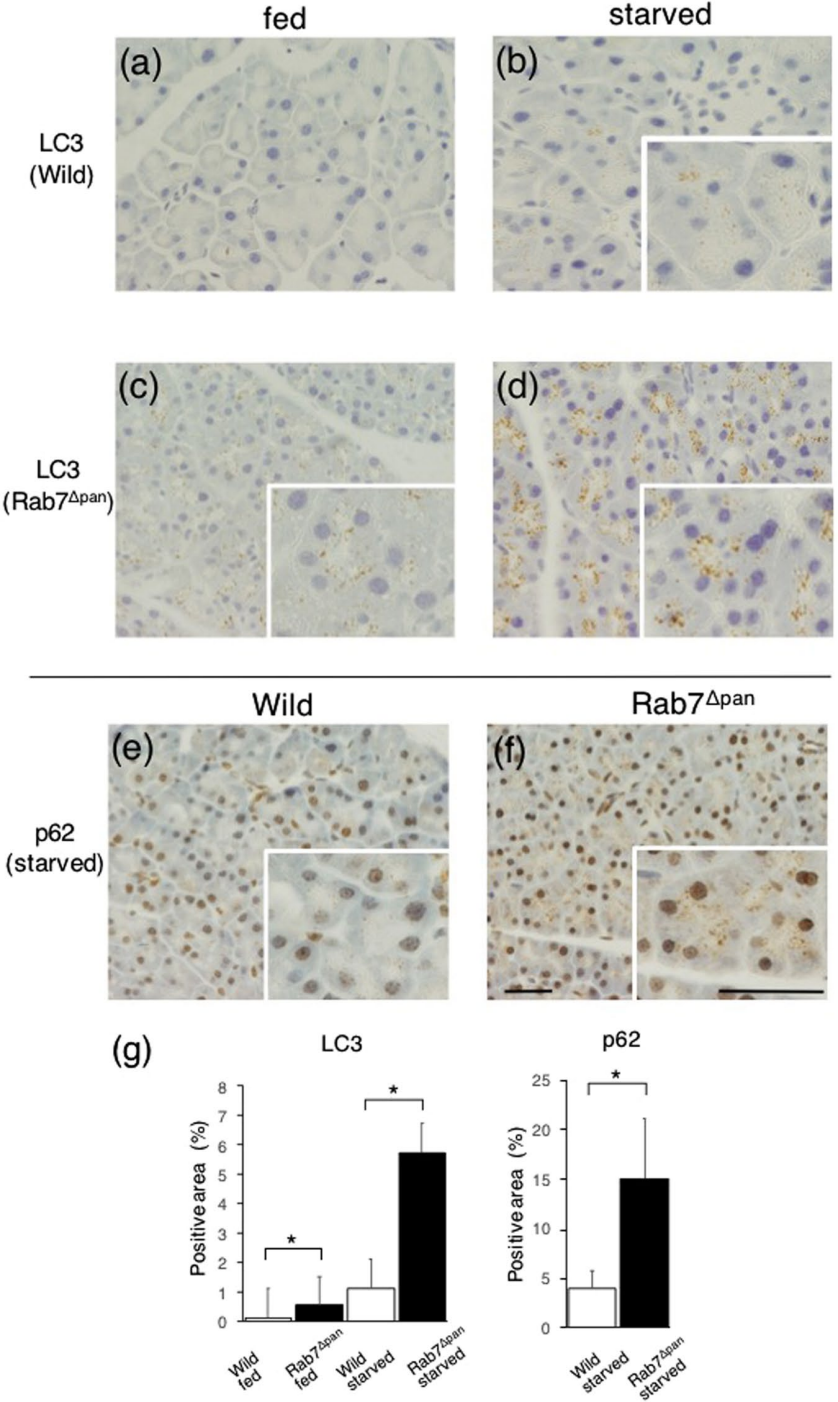
The increased expression of both LC3 and p62 in Rab7^Δpan^ pancreas. IHC of LC3 (**a**–**d**) and p62 (**e**,**f**) was performed using wild (**a**,**b**,**e**) and Rab7^Δpan^ (**c**,**d**,**f**) mouse pancreases. In panels (**b**,**d**,**e** and **f**), the specimens were prepared after 24-h starvation. Scale Bars: 20 µm. (**g**) Quantification of the positive signals in images of immunohistochemistry images of LC3 (left panel) and p62 (right panel).

We then investigated the expression of LC3-II, a marker of autophagic vacuole formation, and p62 (SQSTM1), a major substrate of autophagy, in order to analyze the autophagy function in Rab7^Δpan^ mouse pancreas. As shown in Fig. 2c, LC3-II was expressed even in fed Rab7^Δpan^ mouse pancreas and was strongly expressed in starved Rab7^Δpan^ mouse pancreas (Fig. 2c). In addition, the amount of p62 increased in starved Rab7^Δpan^ mouse pancreas (Fig. 2d). We further analyzed the expression of LC3 and p62 with immunohistochemistry (IHC). As shown in Fig. 3, IHC using anti-LC3 antibody showed little signal in fed wild mouse pancreatic acinar cells (Fig. 3a). Interestingly, however, weak but apparent dot signals were observed in fed Rab7^Δpan^ mouse pancreatic acinar cells (Fig. 3c and g), as well as in those of starved wild mice (Fig. 3b and g). Furthermore, the LC3 dot signals were markedly augmented in starved Rab7^Δpan^ mouse pancreatic acinar cells (Fig. 3d and g). In addition, intense p62 signals were observed via IHC of starved Rab7^Δpan^ mouse pancreatic acinar cells, whereas signals were weak in those of wild mice (Fig. 3e,f and g). These data suggest that autophagy is activated and highly responds to starvation in Rab7^Δpan^ mouse pancreatic acinar cells according to the higher expression of LC3-II, but autophagy flux is impaired in Rab7^Δpan^ mouse pancreatic acinar cells according to the higher expression of p62.

**Figure 4:**
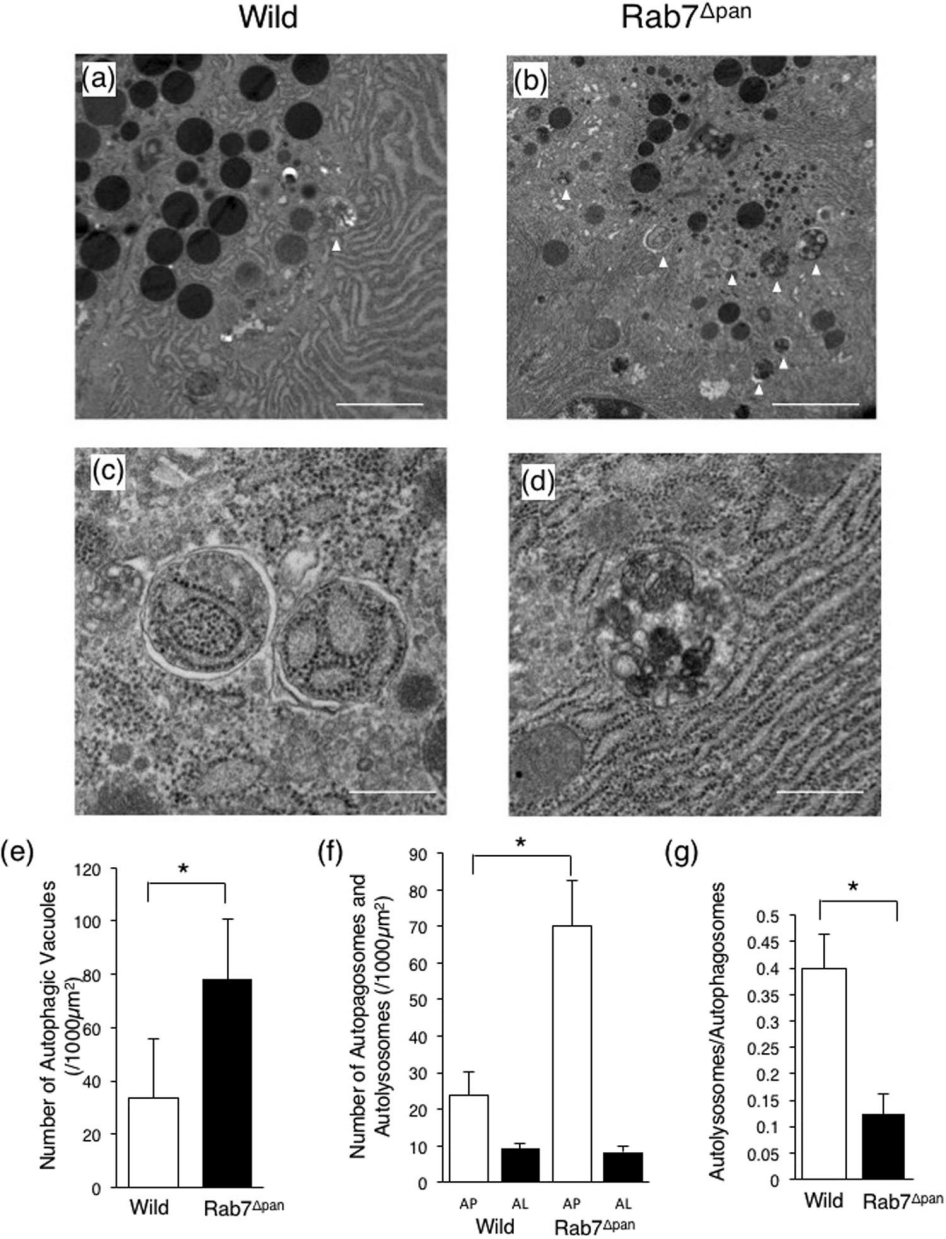
An EM evaluation of autophagic vacuoles induced by 24-h starvation. (**a**,**b**) EM images of wild (**a**) and Rab7^Δpan^ (**b**) mouse pancreatic acinar cells after 24-hour starvation. Arrow heads: autophagic vacuoles. Scale Bars: 2 µm. (**c**,**d**) Representative images of autophagosomes (**c**) and an autolysosome (**d**) formed in starved Rab7^Δpan^ pancreatic acinar cells. Scale Bars: 0.5 µm. (**e**) An EM analysis of autophagic vacuole abundance. (**f**,**g**) An EM comparison of the number of autophagosomes (AP) and autolysosomes (AL) (**f**) and the ratio of autolysosomes/autophagosomes (**g**) formed after 24-h starvation between control and Rab7^Δpan^ mice. For each count, at least 100 images from 3 mice were quantified (**e**,**f**,**g**). *P < 0.01.

To further analyze the autophagy flux impairment in Rab7^Δpan^ mouse pancreatic acinar cells, we performed electron microscopy (EM) to clarify the vacuole characteristics in Rab7^Δpan^ mouse pancreatic acinar cells. EM revealed that many more autophagic vacuoles were formed in pancreatic acinar cells of starved Rab7^Δpan^ mouse than in starved wild mice (Fig. 4a and b). Specifically, the autophagic vacuoles observed in starved Rab7^Δpan^ mouse pancreatic acinar cells were primarily autophagosomes characterized with surrounding double-layer membranes (Fig. 4c), and autolysosomes (Fig. 4d) were markedly decreased in starved Rab7^Δpan^ mouse pancreatic acinar cells (Fig. 4e–g). These data suggest that the autophagy flux in Rab7^Δpan^ pancreatic acinar cells is impaired at the maturation step from autophagosome to autolysosome.

### Aberration of endocytic vesicles in Rab7^Δpan^ pancreatic acinar cells

**Figure 5:**
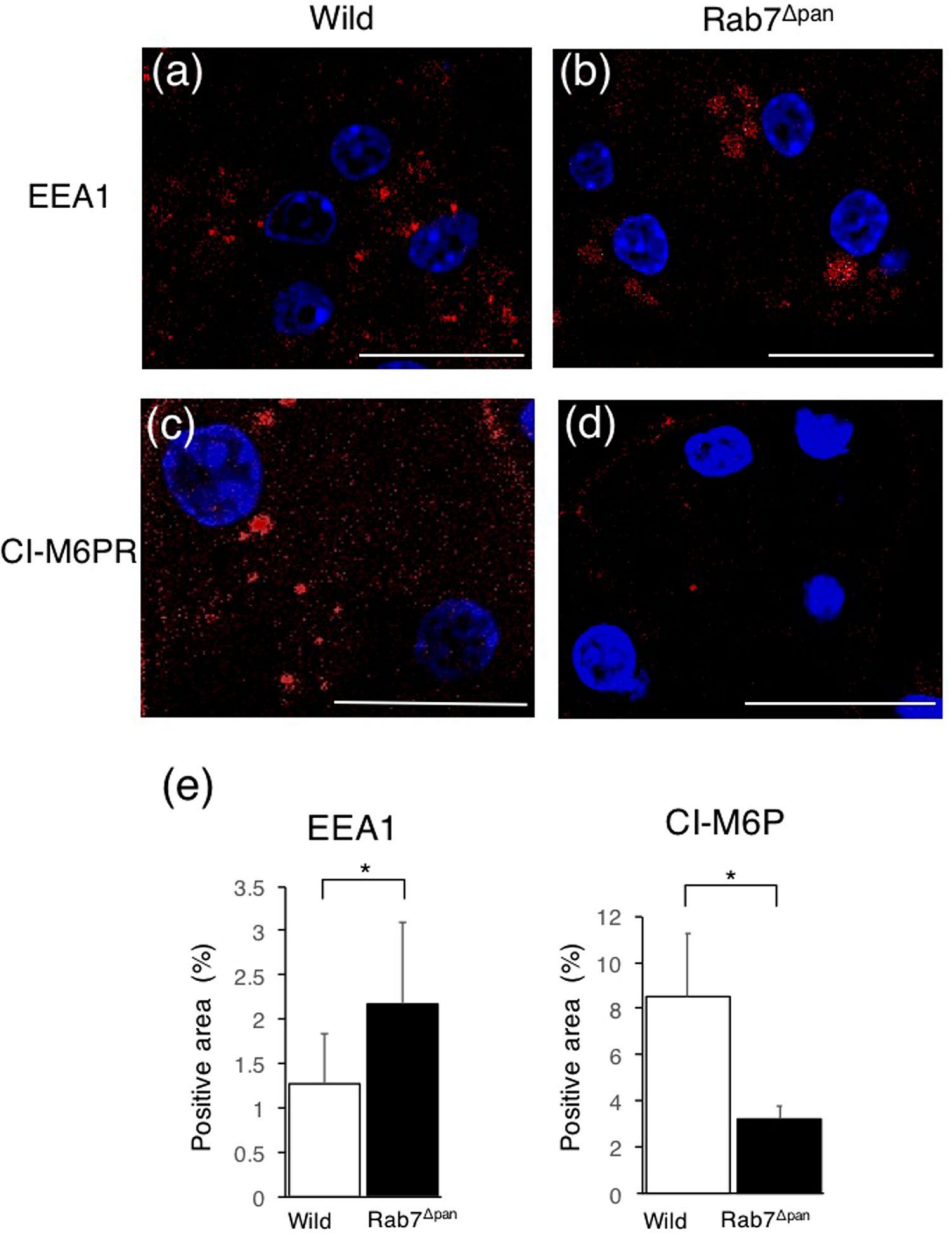
Alteration of the size of early and late endosomes in Rab7^Δpan^ pancreatic acinar cells. Immunofluorescence images of wild (**a**,**c**) and Rab7^Δpan^ (**b**,**d**) pancreases stained with anti-EEA1 (**a**,**b**) or anti-CI-M6PR (**c**,**d**) antibodies (red). DAPI was used for nuclear staining (blue). Bars: 20 µm. (**e**) Quantification of the positive signals in immunofluorescence images of EEA1 (left panel) and CI-M6P (right panel). *P < 0.05.

We next examined the effect of Rab7 disruption on endocytosis in pancreatic acinar cells. Because Rab7 functions in the flux between early and late endosomes^20, 21^, we investigated the morphology of endocytic vesicles using EEA1 and cation-independent mannose6-phospate receptor (CI-MPR) as markers of early and late endosomes, respectively. As shown in Fig. 5, early endosomes were markedly enlarged in Rab7^Δpan^ pancreatic acinar cells compared with those in wild (Fig. 5a,b and e). In contrast, late endosomes were significantly smaller in Rab7^Δpan^ pancreatic acinar cells than in wild mice (Fig. 5c,d and e). These data suggest that Rab7 disruption blocks endocytosis at the flux from early to late endosomes.

### Aberration of lysosomal morphology and the expression of LAMP-1 in Rab7^Δpan^ pancreas

**Figure 6:**
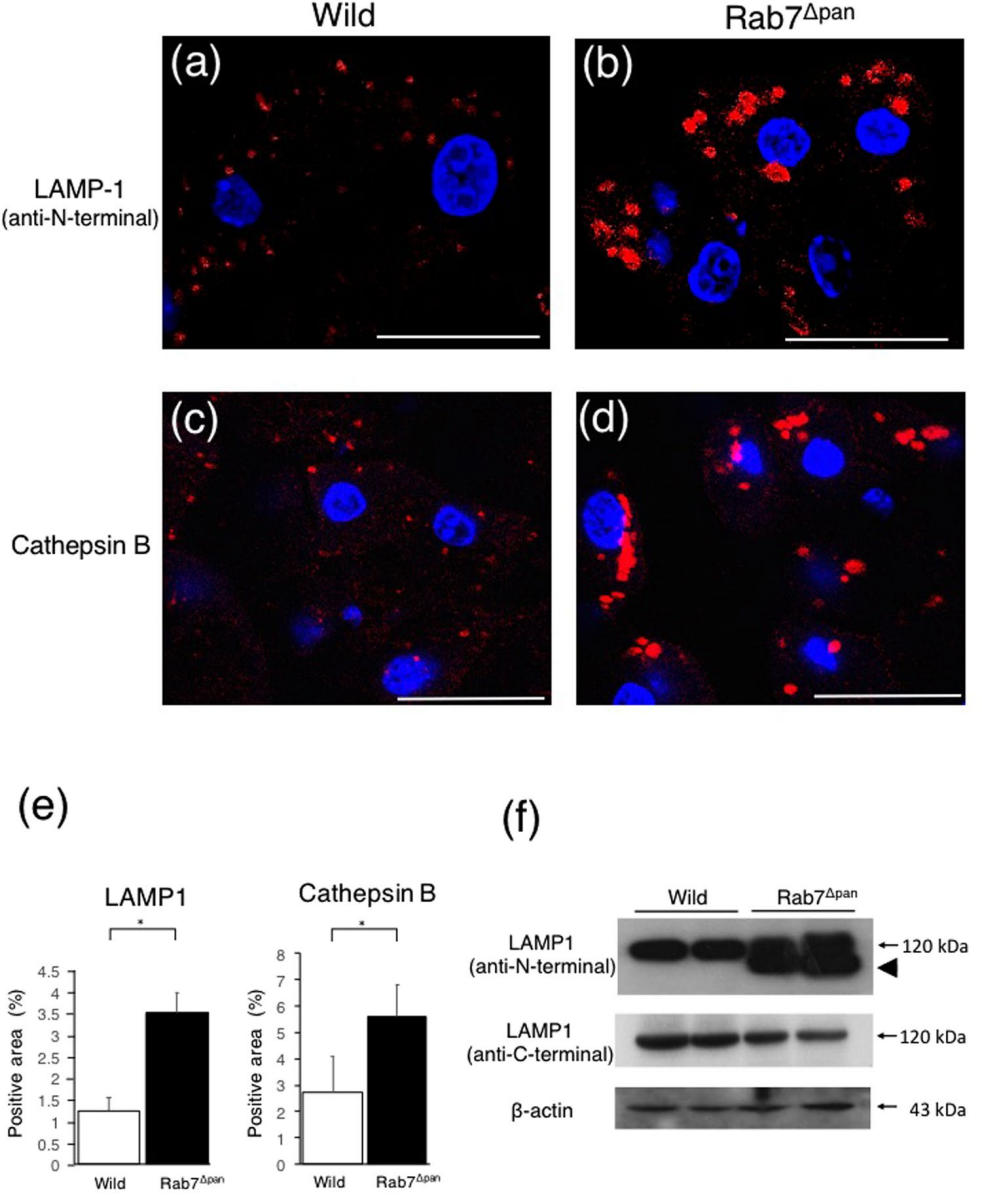
Alteration of the size of lysosomes and the expression of LAMP-1 in in Rab7^Δpan^ pancreatic acinar cells. (**a–d**) Immunofluorescence images of wild (**a**,**c**) and Rab7^Δpan^ (**b**,**d**) pancreases stained with anti-LAMP1(**a**,**b**) or anti-cathepsin B (**c**,**d**) antibodies (red). DAPI was used for nuclear staining (blue). Bars: 20 µm. (**e**) Quantification of the positive signals in immunofluorescence images of LAMP-1 (left panel) and cathepsin B (right panel). *P < 0.05. (**f**) WB of LAMP-1 using total pancreas homogenate of wild and Rab7^Δpan^ mice. An antibody against LAMP-1 N-terminal (top panel) and an antibody against LAMP-1 C-terminal (middle panel) were utilized. Anti-LAMP-1 N-terminal antibody revealed the shifting of intense bands to a lower position (top panel, arrow head) than that of full-length LAMP-1 (120 kDa) in Rab7^Δpan^ pancreas. In contrast, anti-LAMP-1 C-terminal antibody revealed bands at 120 kDa only in both wild and Rab7^Δpan^ pancreas (middle panel). β-actin was used as an internal loading control (bottom panel).

Because both autophagy and endocytosis are driven towards lysosomes and Rab7 has been shown to participate in lysosome biogenesis^14^, we examined lysosomal morphology and the expression of LAMP-1, which is essential for the lysosomal functions^22^. As shown in Fig. 6, immunofluorescence microscopy utilizing LAMP-1 (Fig. 6a and b) and cathepsin B (Fig. 6c and d) as lysosomal markers revealed that lysosomes were enlarged in Rab7^Δpan^ pancreatic acinar cells (Fig. 6a–e). Furthermore, WB showed the increased expression of LAMP-1 in Rab7^Δpan^ (Fig. 6f). Notably, intense bands of LAMP-1 in Rab7^Δpan^ shifted to a slightly lower molecular mass position, suggesting LAMP-1 degradation (Fig. 6f, top panel, arrow head). To confirm this hypothesis, we again performed WB using an anti-LAMP-1 antibody that specifically recognizes the C-terminal short tail. As expected, the antibody recognized the LAMP-1 bands in wild mice and the higher bands in Rab7^Δpan^ pancreas but not the lower bands in Rab7^Δpan^ pancreas (Fig. 6f, middle panel), indicating that LAMP-1 is degraded at its C-terminal in Rab7^Δpan^ pancreas. These data imply that the lysosomal functions in Rab7^Δpan^ pancreatic acinar cells are affected by Rab7 disruption.

### Exacerbation of the Severity of experimental acute pancreatitis in Rab7^Δpan^ pancreas

**Figure 7:**
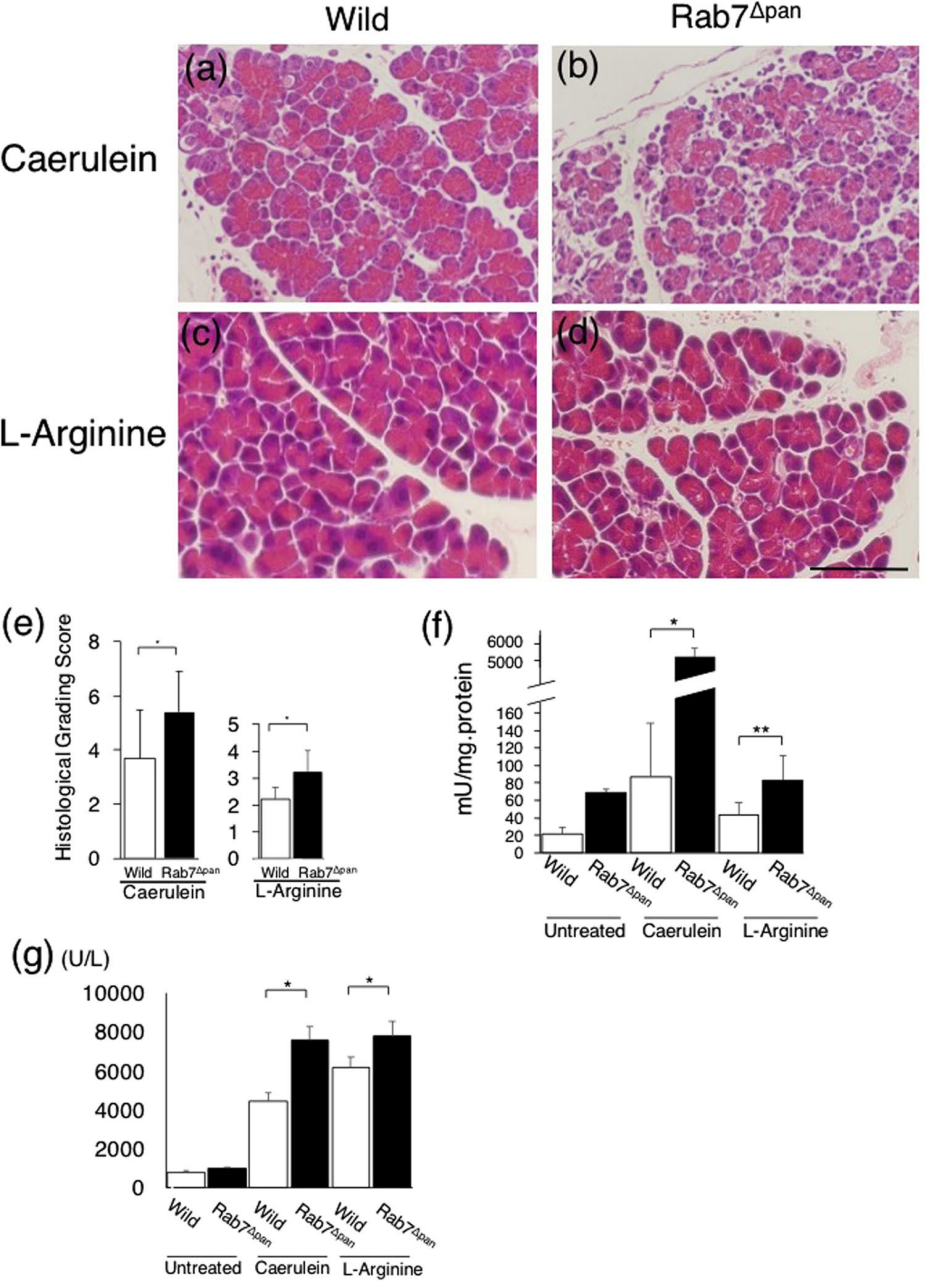
The evaluation of the severity of caerulein- and L-arginine-induced acute pancreatitis. (**a–d**) HE images of wild (**a**,**c**) and Rab7^Δpan^ (**b**,**d**) mouse pancreases with caerulein- (**a**,**b**) or L-arginine (**c**,**d**)-induced acute pancreatitis. Scale Bar: 50 µm. (**e**) Histological grading of the severity of acute pancreatitis based on pancreatic edema, inflammatory cellular infiltration and acinar cell necrosis, as described previously^36^. n = 5. *P < 0.05. (**f**) The intrapancreatic trypsin activity was determined in triplicate. n = 3. *P < 0.01, **P < 0.05 by Student’s *t*-test. (**g**) Serum amylase concentration was determined in triplicate. n = 3. *P < 0.05.

As we found that autophagy, endocytosis, and the lysosomal functions were impaired in pancreatic acinar cells of Rab7^Δpan^ mice, we next investigated the effect of Rab7 deficiency on acute pancreatitis using experimental models of acute pancreatitis induced by caerulein or L-arginine. Histopathological analyses with HE staining showed that pancreatic edema, inflammatory cellular infiltration to the pancreas and pancreatic acinar cell necrosis were more severe in Rab7^Δpan^ pancreas than in wild mouse pancreas (Fig. 7a–d). Histological grading scored by two expert pathologists blinded to the experimental conditions confirmed that the severity of pancreatitis was significantly higher in Rab7^Δpan^ mice than in wild mice (Fig. 7e). In addition, the intrapancreatic trypsin activity and serum amylase concentration were also much higher in Rab7^Δpan^ mice than in wild mice (Fig. 7f and g). In particular, the intrapancreatic trypsin activity was markedly elevated in Rab7^Δpan^ pancreas under the conditions of caerulein-induced acute pancreatitis (Fig. 7f). These data imply that the disruption of Rab7 exacerbates the severity of acute pancreatitis in experimental mouse models. Since the severity of acute pancreatitis was markedly higher in caerulein-treated mice than in L-arginine-treated mice, we focused our further analyses on caerulein-induced acute pancreatitis.

### Autophagy flux is more impaired in Rab7^Δpan^ pancreas

**Figure 8:**
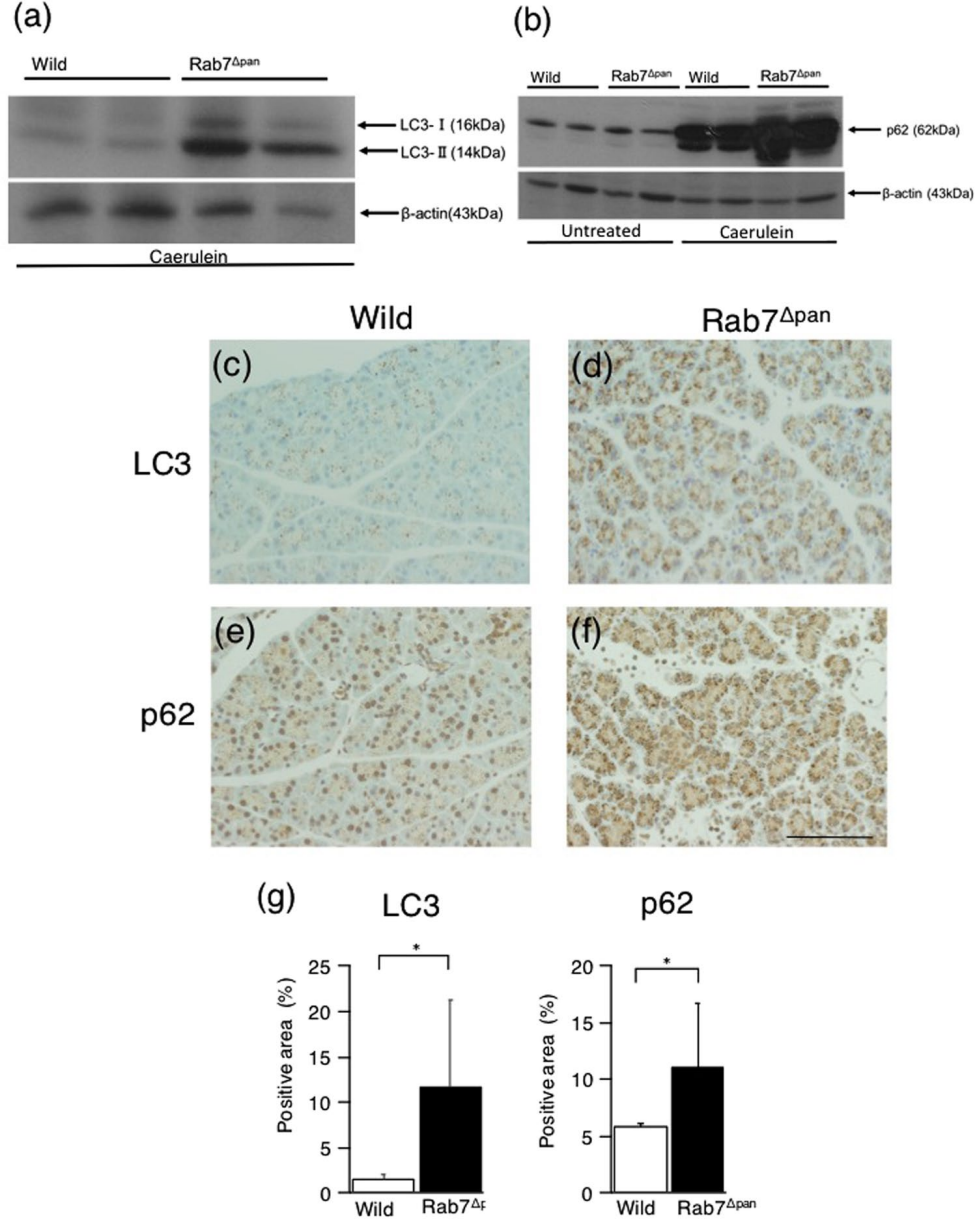
Autophagy in caerulein-induced acute pancreatitis. (**a**,**b**) WB of LC3 (**a**) and p62 (**b**) using total pancreas homogenates of wild and Rab7^Δpan^ mice after (**a**), or before (Untreated) and after (Caerulein) the induction of acute pancreatitis by caerulein (**b**). β-actin was used as an internal loading control. The blots are the representative of two independent experiments with similar results. (**c–f**) IHC of LC3 (**c**,**d**) and p62 (**e**,**f**) was performed using wild (**c**,**e**) and Rab7^Δpan^ (**d**,**f**) mouse pancreases with caerulein-induced acute pancreatitis. Scale Bar: 50 µm. (**g**) Quantification of the positive signals in images of immunohistochemistry of LC3 (left panel) and p62 (right panel). *P < 0.05.

We next compared the autophagic process in caerulein-induced pancreatitis between Rab7^Δpan^ and wild pancreatic acinar cells, as the autophagy flux is reportedly impaired in experimental acute pancreatitis models, even in wild mice^11, 12^. WB showed that although LC3-II was expressed in both wild and Rab7^Δpan^ pancreases during caerulein-induced acute pancreatitis, its expression was much higher in Rab7^Δpan^ mouse pancreas than in wild mouse pancreas (Fig. 8a). Furthermore, the expression of p62 was increased in wild and Rab7^Δpan^ pancreases with caerulein-induced pancreatitis compared to untreated mouse pancreas (Fig. 8b). However, the amount of p62 in pancreas from caerulein-induced pancreatitis mice was higher in Rab7^Δpan^ mice than in wild mice (Fig. 8b). Of note, the sifting of intense bands of p62 to lower molecular positions was also observed in caerulein-treated pancreas, suggesting post translational modified p62 forms. These findings are consistent with those of previous reports that p62 is ubiquitinated and phosphorylated during autophagy process^23, 24^. In addition, IHC revealed that the expression of both LC3 and p62 in pancreatic acinar cells with caerulein-induced acute pancreatitis was higher in Rab7^Δpan^ mice than in wild mice (Fig. 8c–g).

**Figure 9:**
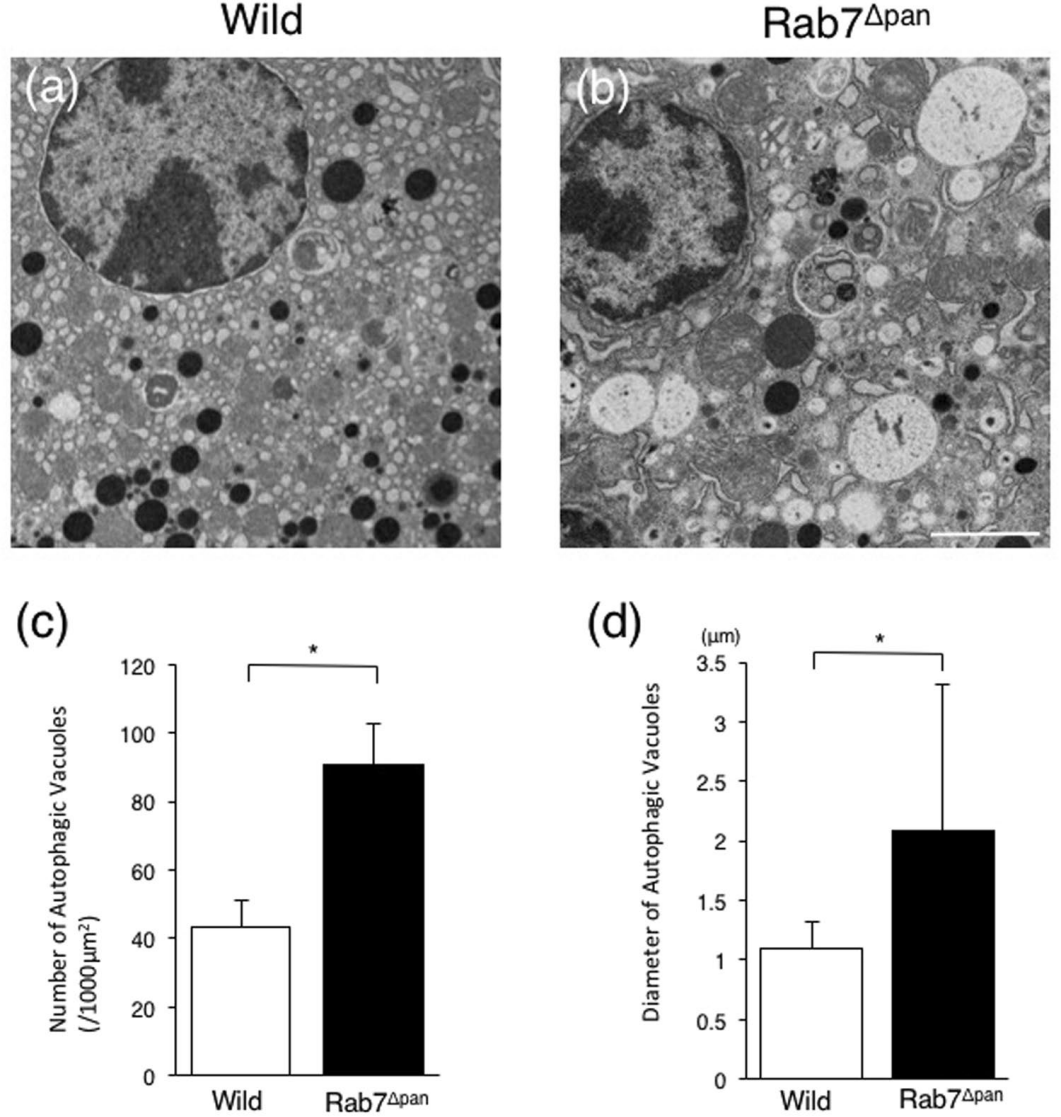
Comparison of autophagic vacuoles formed during caerulein-induced acute pancreatitis between Rab7^Δpan^ and wild mice. (**a**,**b**) EM images of wild (**a**) and Rab7^Δpan^ (**b**) mouse pancreases with caerulein-induced acute pancreatitis. Scale Bar: 2 µm. (**c**,**d**) EM comparison of the number of autophagic vacuoles (**c**) and the diameter of autophagic vacuoles (**d**) formed under conditions of pancreatitis between control and Rab7^Δpan^ mice. For each count, at least 100 images from 3 mice were quantified. *P < 0.05.

**Figure 10:**
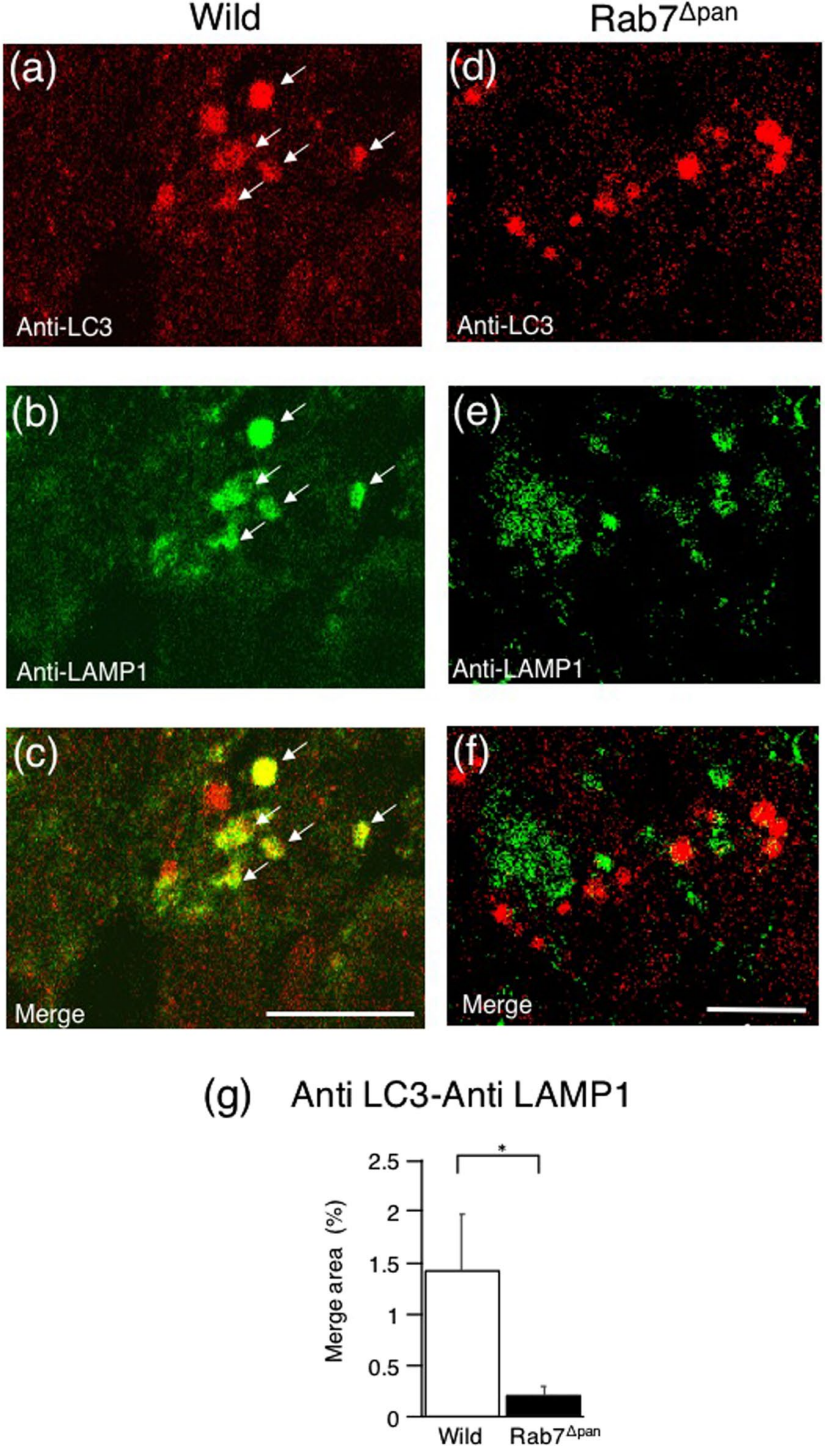
Immunofluorescence images of pancreatic acinar cells with caerulein-induced acute pancreatitis double-stained with anti-LC3 and anti-LAMP-1 antibodies. The yellow signals indicate the colocalization of LC3 and LAMP-1 (arrows). Scale Bars: 10 µm. (**g**) Quantification of the merged signals of LC3 and LAMP1 (yellow signals) in immunofluorescence images. P* < 0.05.

EM further revealed that, in caerulein-induced pancreatitis, autophagic vacuoles were more frequently formed in Rab7^Δpan^ pancreatic acinar cells than in the wild mice (Fig. 9a–c). These data suggest that autophagy is both increasingly activated and increasingly impaired in Rab7^Δpan^ pancreatic acinar cells. In addition, the vacuoles formed in Rab7^Δpan^ pancreatic acinar cells were larger than those in control mice (Fig. 9d). These data imply that the vacuole formation in Rab7^Δpan^ pancreatic acinar cells during caerulein-induced acute pancreatitis differs markedly from that in wild mice. To investigate the differences in the vacuole formation characteristics, we next performed double-staining immunofluorescence microscopy using anti-LC3 and anti-LAMP1 antibodies. In the wild mouse pancreas, LC3 and LAMP-1 were often colocalized to vacuoles (Fig. 10a–c, arrows, and Fig. 10g). In contrast, this colocalization was hardly observed in Rab7^Δpan^ pancreas (Fig. 10d–g). These data suggest that the vacuoles formed in Rab7^Δpan^ pancreas during caerulein-induced acute pancreatitis predominantly possess characteristics of autophagosomes, suggesting that autophagosome maturation to autolysosome is impaired in Rab7^Δpan^ pancreatic acinar cells with caerulein-induced acute pancreatitis.

### Early endosomes are involved in vacuole formation during acute pancreatitis in Rab7^Δpan^

**Figure 11:**
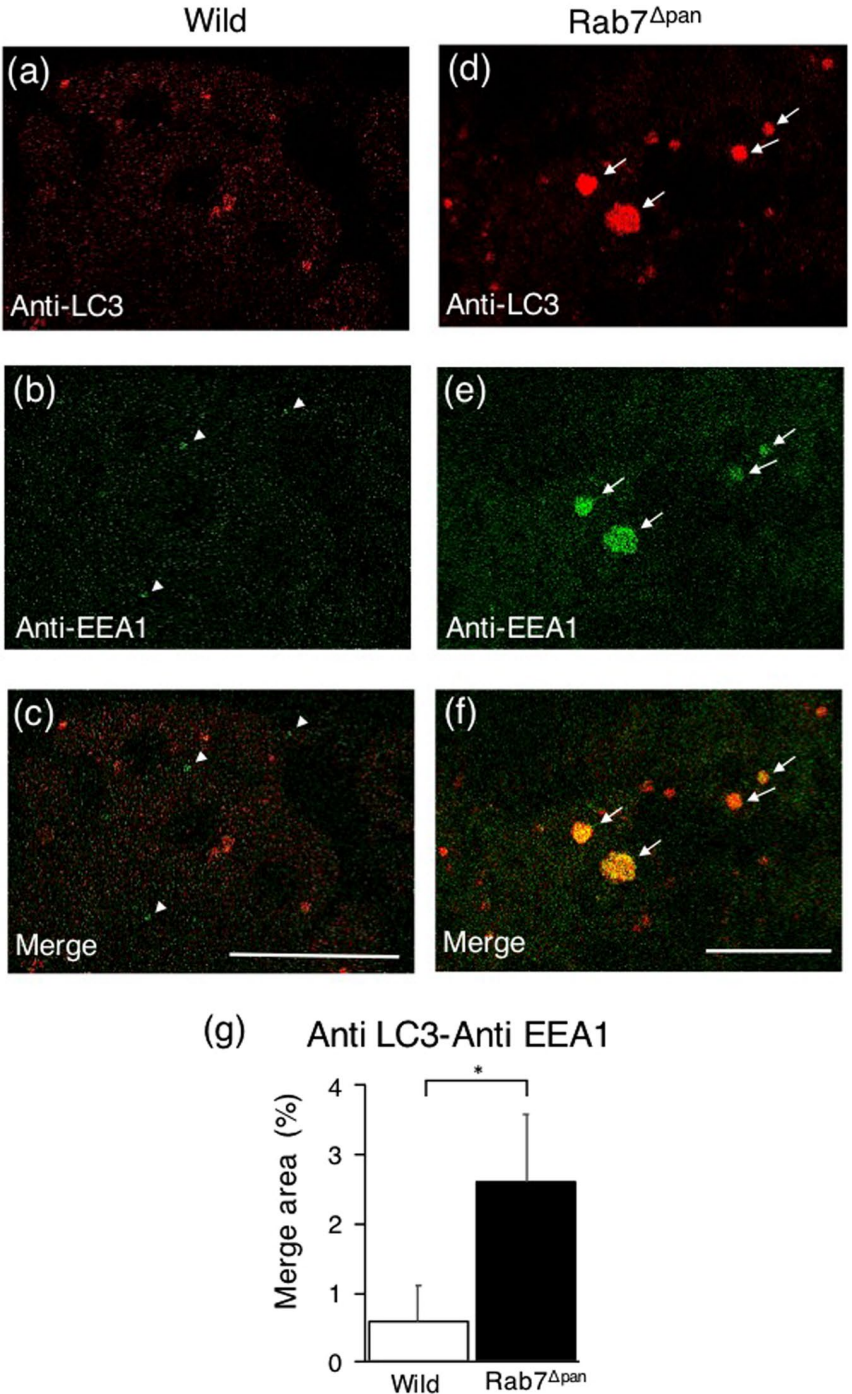
Immunofluorescence images of pancreatic acinar cells with caerulein-induced acute pancreatitis double-stained with anti-LC3 and anti-EEA1 antibodies. The EEA1 signals were small but discernible in wild pancreatic acinar cells (arrowheads). In Rab7^Δpan^ pancreatic acinar cells, the EEA1 signals are highly colocalized with LC3 signals (arrows). Scale Bars: 10 µm. (**g**) Quantification of the merged signals of LC3 and EEA1 (yellow signals) in immunofluorescence images. P* < 0.05.

To further investigate the differences in the characteristics of vacuoles formed during caerulein-induced acute pancreatitis in Rab7^Δpan^ and wild pancreas, we next examined the participation of endocytic vesicles in vacuole formation, as endocytosis is presumed to be involved in the pathogenicity of acute pancreatitis^9^ and the endocytosis process is impaired in untreated Rab7^Δpan^ pancreatic acinar cells (Fig. 5). For this purpose, we performed double-staining immunofluorescence microscopy using anti-LC3 and anti-EEA1 antibodies. Although the EEA1 signals were very low in wild mouse pancreatic acinar cells in caerulein-induced acute pancreatitis (Fig. 11b, arrowheads), these signals rarely colocalized with LC3-positive vacuoles (Fig. 11c, arrow heads, and Fig. 11g). In contrast, EEA1 is tightly colocalized with LC3-positive vacuoles formed in Rab7^Δpan^ pancreatic acinar cells (Fig. 10d–f, arrows, and Fig. 10g). These data indicate that early endosomes are involved in vacuole formation under the conditions of caerulein-induced acute pancreatitis in Rab7^Δpan^ mice.

### Cathepsin B is overexpressed in Rab7^Δpan^ pancreas

**Figure 12:**
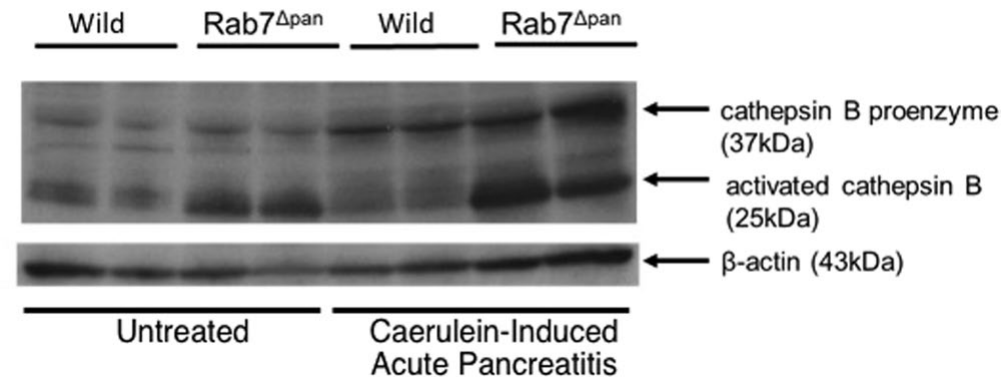
The cathepsin B expression during caerulein-induced acute pancreatitis. WB of cathepsin B using total pancreas homogenates of wild and Rab7^Δpan^ mice before (Untreated) and after the induction of acute pancreatitis by caerulein. β-actin was used as an internal loading control. The blots are the representative of two independent experiments with similar results.

We attempted to elucidate the mechanism underlying the severity of acute pancreatitis in Rab7^Δpan^ mice. To this end, we focused on the extraordinary elevation of intrapancreatic trypsin activity in Rab7^Δpan^ mice during cerulein-induced acute pancreatitis (Fig. 7f). It has been well documented that intrapancreatic trypsin activation primarily contributes to the onset of acute pancreatitis^25, 26^ and lysosomal protease cathepsin B activates trypsin in pancreatic acinar cells during acute pancreatitis^8, 27^. We thus investigated the cathepsin B expression levels in the pancreas. Cathepsin B is originally synthesized as an inactive proenzyme and is proteolytically processed to a single-chain form, and then to a double-chain form (a mature active form)^11, 27^. WB revealed that the signals of the double-chain form were stronger even in untreated Rab7^Δpan^ pancreas than in untreated wild pancreas (Fig. 12). In caerulein-induced acute pancreatitis, the expression of all types of cathepsin B, including proenzyme, and the single- and double-chain forms (mature active form), was further increased in Rab7^Δpan^ pancreas compared with untreated Rab7^Δpan^ pancreas (Fig. 12). In contrast, however, the levels of the double- chain form of cathepsin B were lower in wild pancreas with caerulein-induced acute pancreatitis than in untreated wild pancreas. These data suggest that the marked elevation of intrapancreatic trypsin activity in Rab7^Δpan^ mice during caerulein-induced acute pancreatitis is at least partly attributed to the increased expression of cathepsin B.

## Discussion

In the present study, we demonstrated that the disruption of Rab7 impairs the autophagy flux and endocytosis process, resulting in the exacerbation of the severity of acute pancreatitis.

Autophagy is a sequential intracellular vesicle trafficking process used to degrade cytoplasmic compartments, such as organelles, and proteins using lysosomal enzymes. To date, multiple molecules, such as autophagy-related genes (Atgs), molecular motors and small GTPases, have been shown to be involved in autophagy^1^. In addition to its involvement in physiological cellular responses, autophagy has been clarified to participate in various inflammatory diseases including acute pancreatitis^4^. Hashimoto *et al*. reported that autophagy is involved in the initial intracellular events of acute pancreatitis and Atg5 plays stimulatory roles in acute pancreatitis^10^. Furthermore, because Atg5 functions at the early steps of autophagy, namely at the step of autophagic vacuole formation, the early steps of autophagy are likely involved in the onset and progression of acute pancreatitis^10^. In the late steps of the autophagy, after autophagic vacuole formation, the vacuoles mature from autophagosomes into autolysosomes in order to degrade the engulfed materials using lysosomal enzymes^1^. This maturation occurs primarily through the fusion of autophagosomes and lysosomes. Tanaka *et al*. previously suggested that disturbance of the late steps of autophagy may be involved in vacuole formation in pancreatic acinar cells^28^.

Concerning acute pancreatitis, the functions and aberrations of the late steps of autophagy in pancreatic acinar cells have been studied. Mareninova *et al*. reported that autophagy flux is retarded while autophagic vacuole maturation from an autophagosome to autolysosome was maintained in pancreatic acinar cells in an experimental rat model of caerulein-induced acute pancreatitis^11^. Those authors further showed that autophagy flux impairment induced an intracellular imbalance between lysosomal proteinases cathepsin B and cathepsin L, the major enzymes indispensable for the degradation of engulfed materials at the late steps of autophagy^11^. Cathepsin L degrades both trypsinogen and trypsin, whereas cathepsin B converts trypsinogen to trypsin^11, 27^. Thus, they concluded that the imbalance of enzymatic activities of cathepsin B and cathepsin L in ZG-rich fractions resulted in intracellular trypsin activation in acute pancreatitis^11^. In contrast, we showed in the present study that caerulein-induced acute pancreatitis markedly increased the expression levels of all the three isoforms of the cathepsin B, namely the proenzyme and single- and double-chain forms (Fig. 12). However, consistent with the report by Mareninova *et al*.^11^, the expression of the double-chain form (mature active form) of cathepsin B was slightly decreased in wild mice with acute pancreatitis compared with untreated wild mice (Fig. 12). These data indicate that the cathepsin B expression is highly enhanced in Rab7^Δpan^ pancreas contributing to the increased trypsin activation in Rab7^Δpan^ pancreas during acute pancreatitis.

Regarding the possibility that autophagosome to autolysosome maturation is compromised in acute pancreatitis^8^, Fortunato *et al*. revealed that autophagy is inhibited at the late step of autophagic vacuole maturation from autophagosome to lysosome concomitantly with the depletion of LAMP-2 in pancreatic acinar cells, resulting in the accumulation of autophagic vacuoles in pancreatic acinar cells^12^. The discrepancy between these two reports^11, 12^ on autophagic vacuole maturation might be attributed to the differences in the experimental models of acute pancreatitis and rodent species used. Nonetheless, these data strongly indicated that various aberrations of the late steps of autophagy play critical roles in the onset and progression of acute pancreatitis. However, the precise molecular mechanism underlying the development of acute pancreatitis remains unclear. We therefore conducted experiments in pancreas-specific Rab7 knockout mice in the present study. Notably, we clarified the involvement of the late steps of autophagy in the onset of acute pancreatitis using genetic engineering of a molecule that directly functions in the late steps of autophagy. In this novel *in vivo* model of autophagy impairment, we further demonstrated the aberration of lysosomal functions by showing enlarged lysosomes and an increased LAMP-1 expression with C-terminal degradation. LAMPs are essential proteins required by lysosomes to perform diverse functions. Furthermore, it was recently reported that LAMPs play critical roles in maintaining pancreatic acinar cell homeostasis^29^. We therefore speculate that the lysosomal aberration observed in Rab7^Δpan^ pancreas induces the increased active cathepsin B expression and marked elevation of intrapancreatic trypsin activity during acute pancreatitis.

Rab7 is a small GTPase that functions at the late steps of autophagy, particularly during autophagic vacuole maturation from an autophagosome to an autolysosome^1, 7, 15^. A previous report by Mareninova *et al*. showed that Rab7 protein accumulates in zymogen granules-enriched subcellular fractions in acute pancreatitis^11^, suggesting that Rab7 participates in the onset and progression of acute pancreatitis and prompting us to evaluate the function of Rab7 in acute pancreatitis. In the current study, we confirmed that the autophagic process is inhibited at the step of autophagic vacuole maturation in Rab7^Δpan^ pancreatic acinar cells. Consistent with the report by Mareninova *et al*.^11^, we also observed that, although the autophagy influx was impaired (Fig. 8), autophagic vacuole maturation from autophagosomes to autolysosomes was observed in wild mouse pancreatic acinar cells of caerulein-induced acute pancreatitis (Fig. 10). However, a greater number of intracellular vacuoles, which were determined to be predominantly autophagosomes, were formed in Rab7^Δpan^ pancreatic acinar cells (Fig. 10) in which autophagic vacuole maturation had been inhibited than in wild mouse pancreatic acinar cells. These results are consistent with those of previous reports that the inhibition of autophagosome maturation to autolysosomes increased the LC3-II expression and formation of autophagic vacuoles^2, 12, 28, 30^. Furthermore, the levels of intrapancreatic trypsin activity and pancreatic inflammation were higher in Rab7^Δpan^ mice than wild mice (Fig. 7). In addition, the size of the vacuoles formed in acute pancreatitis was larger in Rab7^Δpan^ mice than in wild mice (Fig. 9), suggesting that caerulein-induced pancreatitis disturbs the autophagic process more severely in Rab7^Δpan^ mice than in wild mice, as shown previously^5, 8^. Taken together, these data indicated that the blockade of autophagic vacuole maturation from autophagosome to autolysosome by Rab7 disruption exacerbates the severity of acute pancreatitis.

In addition to autophagy, Rab7 plays crucial roles in endocytosis^20, 21^, especially at late stages of endosome flux and maturation. Thus, consistent with previous reports^31, 32^, we may reasonably presume that the early endosome enlargement observed in untreated Rab7^Δpan^ pancreatic acinar cells (Fig. 5) was due to the blockade of the flux from early to late endosomes by Rab7 disruption. In contrast, the depletion of late endosomes observed in the current study (Fig. 5) is inconsistent with these previous reports^31, 32^. This discrepancy might be due to the differences in the cell types utilized in the experiments. Interestingly, our present data showed that EEA1 is colocalized with LC3-positive vacuoles formed in Rab7^Δpan^ pancreatic acinar cells during acute pancreatitis (Fig. 11). In this respect, Messenger *et al*. recently demonstrated that altered trafficking from early to late endosomes led to the onset of acute pancreatitis^9^. Their data support our hypothesis that the blockade of the endosome flux from early to late endosomes by Rab7 disruption might enhance vacuole formation during acute pancreatitis by involving early endosomes in the vacuole formation mechanism.

In conclusion, we herein showed that Rab7 disruption exacerbates the severity of acute pancreatitis, indicating that Rab7 plays a protective role in acute pancreatitis. These observations therefore provide valuable insights into the development of novel strategies for the treatment of acute pancreatitis.

## Materials and Methods

### Antibodies

The primary antibodies used in this study were: rabbit anti-Rab7 (for IHC: ab 137029; Abcam, Cambridge, UK), chicken anti-Rab7 (for WB), rabbit anti-LC3 (for WB: PM036; MBL, Nagoya, Japan) (for IHC: #3868; Cell Signaling Technology), guinea pig anti-p62 (PM066; MBL), rat anti-N-terminal of lysosomal-associated membrane protein (LAMP)-1 (553792; BD Biosciences, San Jose, CA, USA) and rabbit anti-C-terminal of LAMP-1 (ab24170; Abcam), mouse anti-cathepsin B (AF965; R&D Systems, Minneapolis, MN, USA), rabbit anti-EEA1 (#2411; Cell Signaling Technology), mouse anti-CI-MPR (NB300–514; Novus, Littleton, CO, USA) and goat anti-actin (sc-1616; Santa Cruz Biotechnology, Dallas, TX, USA) immunoglobulins (IgGs). The secondary antibodies used in this study were: horseradish peroxidase-conjugated goat anti-guinea pig (Cosmo Bio, Tokyo, Japan), donkey anti-rabbit, anti-chicken and anti-mouse IgGs, and FITC- or Cy3-conjugated donkey anti-rabbit, anti-mouse and anti-rat IgGs (Jackson ImmunoResearch, West Grove, PA, USA).

### Generation of pancreas-specific Rab7-deficient mice

Rab7^*flox/flox*^ mice (C57bl6/J) were generated as previously reported^33^. Rab7^*flox/flox*^ mice were crossed with Ptf1a-Cre mice that were kindly provided by Professor Yoshiya Kawaguchi at Kyoto University^34^. Offspring carrying Ptf1a-Cre and two copies of the floxed Rab7 allele (Ptf1a-Cre Rab7^flox/flox^) were used in this study as homozygous mutant (Rab7^Δpan^) mice. In addition, littermates carrying two copies of the Rab7 allele, but not Ptf1a-Cre (Rab7^flox/flox^), were used as wild-type (wild) mice. These genotypes were confirmed via polymerase chain reaction using the following primer pairs: Rab7, sense: 5′-ACCTGGAAGAGTGAACCAAGGG- TCAGCATG-3′, antisense: 5′-ACCCCTGCCTGGGATTTTGGTCCTGGATTC-3′, Ptf1a-Cre, sense: 5′-ACCTGAAGATGTTCGCGATTATCT-3′, antisense: 5′-ACC- GTCAGTACGTGAGATATCTT-3′. All experiments were carried out with male Rab7^Δpan^ and wild mice. All experiments using mice were approved by and were carried out in accordance with the guideline approved by the Institutional Animal Care and Use Committee of Akita University.

### Caerulein- and L-arginine-induced pancreatitis

Acute pancreatitis was induced in age-matched 8- to 12-week-old male mice using caerulein or L-arginine. Caerlein-induced pancreatitis was triggered by injecting 50 µg/kg (body weight) caerulein (Sigma-Aldrich) intraperitoneally 10 times hourly. The mice were euthanized 1 h after the last injection. L-arginine-induced acute pancreatitis was triggered by injecting 4 g/kg (body weight) L-arginine (Sigma-Aldrich) intraperitoneally twice hourly. The mice were euthanized 72 h after the last injection. Three independent experiments were conducted for all experiments.

### Western blotting

Briefly, samples of 10 to 40 µg proteins were loaded onto 8–14% sodium dodecyl sulphate-polyacrylamide electrophoresis gels and run at 200 v. After gel electrophoresis, the proteins were transferred to nitrocellulose membranes at 60 v for 4 h, except for LC3 blotting, for which the proteins were transferred to PVDF membranes. WB was performed as previously described^35^ using an enhanced chemiluminescence reagent to visualize the secondary antibodies.

### Immunohistochemistry, histology and electron microscopy

Excised pancreatic tissues were formalin-fixed and paraffin-embedded or frozen sections were used for immunohistochemistry. Immunofluorescence microscopy was performed as described previously^19^. In some experiments, 4′,6-diamidino-2-phenylindole (DAPI) (Sigma-Aldrich) was used for nuclear staining. The samples were examined under a Zeiss confocal microscope LSM780 (Zeiss Co., Oberkochen, Germany). The histological scores of the severity of caerulein-induced acute pancreatitis were determined by evaluating hematoxylin-eosin (HE) stained specimens by two expert pathologists who had been blinded to the experimental information, in accordance with the criteria previously described by Laethem *et al*.^36^. For the scoring criteria, pancreatic edema, inflammatory cell infiltration and acinar necrosis were each scored from 0 to 3, and the total score indicated the severity of caerulein-induced pancreatitis. The images of immunohistochemistry and immunofluorescence microscopy were quantitatively analyzed using the NIH Image J software program^37^. The width of the positive signals was expressed as a percent of the whole width of the analyzed field. EM was carried out as described previously^38^.

### Trypsin Activity

The trypsin activity was measured as previously described^10^. Briefly, the trypsin activity of pancreatic tissue homogenates in ice-cold buffer was fluorometrically determined using Boc-Gln-Ala-Arg-MCA (Bechem, Offenburg, Germany) as the substrate in accordance with the method described by Kawabata *et al*.^39^.

### Statistical analysis

All data are presented as the means ± standard deviation. The statistical significance of the values was analyzed by Student’s *t*-test for comparisons between two groups and by an analysis of variance for comparisons among more than two groups unless otherwise indicated. A P-value of < 0.05 was considered to be significant.

## References

[1] Eskelinen EL (2005). Maturation of autophagic vacuoles in Mammalian cells. Autophagy.

[2] Ferraro E, Cecconi F (2007). Autophagic and apoptotic response to stress signals in mammalian cells. Arch. Biochem. Biophys..

[3] Gukovsky I, Li N, Todoric J, Gukovskaya A, Karin M (2013). Inflammation, autophagy, and obesity: Common features in the pathogenesis of pancreatitis and pancreatic cancer. Gastroenterology.

[4] Czaja MJ (2011). Functions of autophagy in hepatic and pancreatic physiology and disease. Gastroenterology.

[5] Mehrpour M, Esclatine A, Beau I, Codogno P (2010). Autophagy in health and disease. 1. Regulation and significance of autophagy: an overview. Am. J. Physiol. Cell Physiol.

[6] Somsel Rodman J, Wandinger-Ness A (2000). Rab GTPases coordinate endocytosis. J. Cell Sci..

[7] Hyttinen JMT, Niittykoski M, Salminen A, Kaarniranta K (2013). Maturation of autophagosomes and endosomes: A key role for Rab7. Biochim. Biophys. Acta - Mol. Cell Res.

[8] Gukovskaya, a. S. & Gukovsky, I. Autophagy and pancreatitis. *AJP Gastrointest. Liver Physiol*. **303**, G993–G1003 (2012). 10.1152/ajpgi.00122.2012 PMC3517664 22961802

[9] Messenger SW (2015). Early to late endosome trafficking controls secretion and zymogen activation in rodent and human pancreatic acinar cells. Cell. Mol. Gastroenterol. Hepatol.

[10] Hashimoto D (2008). Involvement of autophagy in trypsinogen activation within the pancreatic acinar cells. J Cell Biol.

[11] Mareninova, O. a. *et al*. Impaired autophagic flux mediates acinar cell vacuole formation and trypsinogen activation in rodent models of acute pancreatitis. *J. Clin. Invest*. **119**, 3340–3355 (2009). 10.1172/JCI38674 PMC2769194 19805911

[12] Fortunato F (2009). Impaired autolysosome formation correlates with lamp-2 depletion: role of apoptosis, autophagy, and necrosis in pancreatitis. Gastroenterology.

[13] Ao X, Zou L, Wu Y (2014). Regulation of autophagy by the Rab GTPase network. Cell Death Differ.

[14] Bucci C, Thomsen P, Nicoziani P, McCarthy J, van Deurs B (2000). Rab7: a key to lysosome biogenesis. Mol. Biol. Cell.

[15] Jäger S (2004). Role for Rab7 in maturation of late autophagic vacuoles. J. Cell Sci..

[16] Jin RU, Mills JC (2014). RAB26 coordinates lysosome traffic and mitochondrial localization. J. Cell Sci..

[17] Ohnishi H, Samuelson LC, Yule DI, Ernst SA, Williams JA (1997). Overexpression of Rab3D enhances regulated amylase secretion from pancreatic acini of transgenic mice. J Clin Invest.

[18] Chen X (2004). Rab27b localizes to zymogen granules and regulates pancreatic acinar exocytosis. Biochem. Biophys. Res. Commun..

[19] Ohnishi H (1999). Involvement of Rab4 in regulated exocytosis of rat pancreatic acini. Gastroenterology.

[20] Vitelli R (1997). Role of the small GTPase RAB7 in the late endocytic pathway. J. Biol. Chem..

[21] Feng Y, Press B, Wandinger-Ness A (1995). Rab 7: An important regulator of late endocytic membrane traffic. J. Cell Biol..

[22] Saftig P, Klumperman J (2009). Lysosome biogenesis and lysosomal membrane proteins: trafficking meets function. Nat. Rev. Mol. Cell Biol..

[23] Manley S, Willialms JA, Ding WX (2013). The role of p62/SQSTM1 in liver physiology and pathogenesis. Exp. Biol. Med..

[24] Matsumoto G, Wada K, Okuno M, Kurosawa M, Nukina N (2011). Serine 403 phosphorylation of p62/SQSTM1 regulates selective autophagyc clearance of ubiquitinated proteins. Mol. Cell.

[25] Dawra R (2011). Intra-acinar trypsinogen activation mediates early stages of pancreatic injury but not inflammation in mice with acute pancreatitis. Gastroenterology.

[26] Gaiser S (2011). Intracellular activation of trypsinogen in transgenic mice induces acute but not chronic pancreatitis. Gut.

[27] Reiser J, Adair B, Reinheckel T (2010). Specialized roles for cysteine cathepsins in health and disease. J. Clin. Invest..

[28] Tanaka Y (2000). Accumulation of autophagic vacuoles and cardiomyopathy in LAMP-2-deficient mice. Nature.

[29] Mareninova OA (2015). Lysosome associated membrane proteins maintain pancreatic acinar cell homeostasis: LAMP-2 deficient mice develop pancreatitis. Cell. Mol. Gastroenterol. Hepatol.

[30] Tanida I, Minematsu-Ikeguchi N, Ueno T, Kominami E (2005). Lysosomal turnover, but not a cellular level, of endogenous LC3 is a marker for autophagy. Autophagy.

[31] Press B, Feng Y, Hoflack B, Wandinger-Ness A (1998). Mutant rab7 causes the accumulation of cathepsin D and cation- independent mannose 6-phosphate receptor in an early endocytic compartment. J. Cell Biol..

[32] Girard E (2014). Rab7 is functionally required for selective cargo sorting at the early endosome. Traffic.

[33] Kawamura N (2012). Delivery of endosomes to lysosomes via microautophagy in the visceral endoderm of mouse embryos. Nat. Commun..

[34] Kawaguchi Y (2002). The role of the transcriptional regulator Ptf1a in converting intestinal to pancreatic progenitors. Nat. Genet..

[35] Suzuki J (2003). Involvement of syntaxin 7 in human gastric epithelial cell vacuolation induced by the Helicobacter pylori-produced cytotoxin VacA. J. Biol. Chem..

[36] Van Laethem JL (1995). Interleukin 10 prevents necrosis in murine experimental acute pancreatitis. Gastroenterology.

[37] Schneider CA, Rasband WS, Eliceiri KW (2012). NIH Image to ImageJ: 25 years of image analysis. Nature Methods.

[38] Mashima H (2011). Interferon regulatory factor-2 regulates exocytosis mechanisms mediated by SNAREs in pancreatic acinar cells. Gastroenterology.

[39] Kawabata S (1988). Highly sensitive peptide-4-methylcoumaryl-7-amide substrates for blood-clotting proteases and trypsin. Eur. J. Biochem..

